# Craniocervical and Cervical Spine Features of Patients with Temporomandibular Disorders: A Systematic Review and Meta-Analysis of Observational Studies

**DOI:** 10.3390/jcm9092806

**Published:** 2020-08-30

**Authors:** Ferran Cuenca-Martínez, Aida Herranz-Gómez, Beatriz Madroñero-Miguel, Álvaro Reina-Varona, Roy La Touche, Santiago Angulo-Díaz-Parreño, Joaquín Pardo-Montero, Tamara del Corral, Ibai López-de-Uralde-Villanueva

**Affiliations:** 1 Departamento de Fisioterapia, Centro Superior de Estudios Universitarios La Salle, Universidad Autónoma de Madrid, 28023 Madrid, Spain; fecuen2@gmail.com (F.C.-M.); aiheg@campuslasalle.es (A.H.-G.); bmadmi@campuslasalle.es (B.M.-M.); 201004657@campuslasalle.es (Á.R.-V.); joaquinp@lasallecampus.es (J.P.-M.); tamaradelcorral@gmail.com (T.d.C.); 2 Motion in Brains Research Group, Institute of Neurosciences and Movement Sciences (INCIMOV), Centro Superior de Estudios Universitarios La Salle, Universidad Autónoma de Madrid, 28023 Madrid, Spain; sangulo@ceu.es (S.A.-D.-P.); ibai.uralde@gmail.com (I.L.-d.-U.-V.); 3 Instituto de Neurociencia y Dolor Craneofacial (INDCRAN), 28008 Madrid, Spain; 4 Facultad de Medicina, Universidad CEU San Pablo, 28003 Madrid, Spain; 5 Department of Radiology, Rehabilitation and Physiotherapy, Faculty of Nursing, Physiotherapy and Podiatry, Complutense University of Madrid, 28040 Madrid, Spain

**Keywords:** temporomandibular disorders, cervical spine, neck disability, jaw disability, sensory-motor variables, motor control

## Abstract

To assess neck disability with respect to jaw disability, craniocervical position, cervical alignment, and sensorimotor impairments in patients with temporomandibular disorders (TMD), a systematic review and meta-analysis of observational studies trials were conducted. The meta-analysis showed statistically significant differences in the association between neck disability and jaw disability (standardized mean difference (SMD), 0.72 (0.56–0.82)). However, results showed no significant differences for cervical alignment (SMD, 0.02 (−0.31–0.36)) or for the craniocervical position (SMD, −0.09 (−0.27–0.09)). There was moderate evidence for lower pressure pain thresholds (PPT) and for limited cervical range of motion (ROM). There was limited evidence for equal values for maximal strength between the patients with TMD and controls. There was also limited evidence for reduced cervical endurance and conflicting evidence for abnormal electromyographic (EMG) activity and motor control in TMD patients. Results showed a clinically relevant association between cervical and mandibular disability in patients with TMD. Regarding sensory-motor alterations, the most conclusive findings were observed in the reduction of PPT and cervical ROM, with moderate evidence of their presence in the patients with TMD. Lastly, the evidence on impaired motor control and cervical EMG activity in patients with TMD was conflicting.

## 1. Introduction

Temporomandibular disorders (TMD) include a set of musculoskeletal disorders involving the temporomandibular joint (TMJ), masticatory musculature, and associated orofacial structures [1]. TMD is the leading cause of chronic nonodontogenic orofacial pain [2].

Pain in the temporomandibular region occurs in approximately 10% of the population older than 18 years and is more prevalent in young and middle-aged adults [3]. In addition, the associated signs and symptoms are more common and severe in women than in men [3,4]. Patients with TMD often experience orofacial pain, especially in the periauricular and temporal area. The pain intensity is directly related to the mandibular activity and, therefore, increases with chewing, and speech and can even hinder these actions [5].

TMD might be related to jaw range of motion and joint noises [6]. When the masticatory musculature is affected, its contraction, palpation, and stretching can increase the perception of pain [6,7]. Patients have been reported to limit their mouth opening to prevent such pain from reoccurring [6]. TMD is also present as joint noises, described as clicks, and is associated with abnormal mandibular dynamics, at times producing pain, the sensation of blockage, and even mandibular block [7]. In addition, 60% of patients with TMD have been found to present depressive symptoms, and 21.8% have presented high levels of pain-associated disability [8].

Studies have found an association between the signs and symptoms in the temporomandibular and cervical regions [9], as well as changes in the isometric strength of cervical flexors according to the bite position of patients with TMD [10]. Psychosocial abnormalities have also been reported, such as a higher rate of disability in the cervical region of patients with TMD [11]. There is evidence that the craniomandibular region and upper cervical spine are related from an anatomical, biomechanical, and neurophysiological standpoint [12,13]. At the neurophysiological level, the afferences of trigeminal and cervical neurons converge in the cervical trigeminal complex, located in the brainstem, integrating nociceptive signals from both regions [13]. TMD often has high chronicity rates, and these findings are compatible with a central sensitization process [14]. The functional relationships between the two regions need to be systematically evaluated to assess a number of key variables, such as cervical disability and its relationship with the disability produced in TMD, as well as other variables of a sensory-motor nature. A number of reviews have been conducted, such as the one by Armijo-Olivo et al. [15]; however, the present study aimed to evaluate a larger set of variables in TMD and their relationship with the cervical region, to improve their assessment and interventions and to potentially minimize the consequences of TMD.

TMD is one of the most common musculoskeletal conditions that generate disability [16]. Manfredini et al. [17] suggested that the psychosocial sphere had a bigger impact on TMD pain-related disability than the physical one. TMD pain-related disability is influenced by the following factors related to the pain experience: treatment-seeking behavior, pain duration, depression, and somatization. A total of 21.8% of patients with TMD have been found to present high levels of pain-associated disability [8,17]. This subpopulation is more likely to experience greater pain intensity and functional impairment [18]. Accordingly, patients’ activities of daily living, sleep patterns, and quality of life can be negatively affected [16,19].

The main objective of the present systematic review and meta-analysis was, therefore, to assess the behavior of neck disability with respect to TMJ disability, craniocervical position, and cervical spine alignment in patients with TMD. We also assessed sensory-motor impairments, such as the pain pressure threshold in the craniocervical region, cervical spine range of motion, cervical strength, electromyographic activity in the cervical muscles, and cervical motor control in the cervical region of patients with TMD. 

## 2. Methods

This systematic review and meta-analysis were performed according to the Preferred Reporting Items for Systematic Reviews and Meta-analysis (PRISMA) guidelines described by Moher et al. [20]. The protocol of this meta-analysis was registered in an international register prior to starting the review (PROSPERO, CRD42020159433).

### 2.1. Study Selection

We included studies in the systematic review and meta-analysis if they met the following criteria: (1) cross-sectional design, either cohort or case-control studies; (2) adult patients diagnosed with TMD (mixed, myogenic, or arthrogenous); (3) reported somatosensory, motor, and disability variables of the cervical spine. Studies were excluded if they included patients with primary headaches or non-specific chronic neck pain.

### 2.2. Search Strategy

The search was conducted by two independent reviewers using the same methods; any differences that emerged during this phase were resolved by consensus. Reference sections from original studies were screened manually.

We conducted a search of observational and comparative studies that included cohort, case-control, and cross-sectional studies using MEDLINE (from 1950 to November 2019), PEDro (from 1950 to November 2019), CINAHL (from 1982 to November 2019), and Google Scholar. The last search was performed in November 2019 (19th). 

To perform the database search, we employed the following strategy: (“temporomandibular joint disorders”[MeSH Terms] AND (“neck pain”[MeSH Terms] OR (“neck”[All Fields] AND “pain”[All Fields]) OR “neck pain”[All Fields] OR “Cervical Vertebrae”[MeSH Terms]) AND (“range of motion”[MeSH Terms] OR “Disability Evaluation”[MeSH Terms] OR “posture”[MeSH Terms] OR “muscle strength”[MeSH Terms] OR “muscle strength dynamometer”[MeSH Terms]) AND (Observational Study[ptyp] OR Comparative Study[ptyp])). This strategy was combined with the following free terms and descriptors: ‘craniocervical posture’, ‘cervical spine alignment’, ‘pressure pain threshold’, ‘cervical strength’, ‘cervical motor control’, ‘disability’.

In addition, bibliographic references of identified publications and published bibliographic reviews were searched by hand for potentially relevant articles.

### 2.3. Selection Criteria and Data Extraction

Two independent reviewers (AHG and BMM) performed the first phase, assessing the relevance of the studies. This first analysis was performed based on information from each study’s title, abstract, and keywords. If the abstracts did not contain sufficient information, the full text was reviewed. During the second phase, we reviewed the full text and checked whether the studies met all of the inclusion criteria. A third reviewer (ARV) acted as a mediator when there were differences between the two reviewers, with the 3 reviewers conducting a consensus [21]. The data described in the results were extracted by means of a structured protocol that ensured the most relevant information from each study was obtained.

### 2.4. Methodological Quality Assessment

We assessed the methodological quality of the selected cohort, cross-sectional, and case-control studies using the modified version of the Newcastle-Ottawa Quality Assessment Scale (NOS) [22]. NOS is appropriate for reviews involving a large number of studies because of its brevity, and it presents moderate inter-rater reliability [23]. The NOS scores 3 criteria with a range of 0 to 4 stars: grade selection of participants, assessment of exposures, outcomes, and comparability, and control of confounding variables, based on 9 questions. The tallied stars provide 4 categories of study quality: (1) poor, 0 to 3 stars; (2) fair, 4 to 5 stars; (3) good, 6 to 7 stars; (4) excellent, 8 to 9 stars [24]. For the analysis of the methodological quality of the cross-sectional studies, we used the NOS modifications proposed by Fingleton et al. [25] with only 3 items: (1) 3/3 was considered good quality; (2) 2/3 was fair; (3) 1/3 was poor quality.

Two independent reviewers examined the quality of the selected studies using the same methods; disagreements between the reviewers were resolved by a consensus that included mediation by a third reviewer. The inter-rater reliability was determined using the Kappa coefficient: (1) κ > 0.7 meant a high level of agreement between the assessors; (2) κ = 0.5–0.7 meant a moderate level of agreement; (3) κ < 0.5 meant a low level of agreement [26].

### 2.5. Qualitative Analysis

For the qualitative analysis of the selected observational studies, we employed an adaptation of the classification criteria provided by van Tulder et al. [21] for randomized controlled trials. The results were categorized into 5 levels depending on the methodological quality: (1) strong evidence, consistent among multiple high-quality case-control/cohort/cross-sectional studies (at least 3); (2) moderate evidence, consistent findings from multiple low-quality case-control/cohort/cross-sectional studies and/or one high-quality case-control/cohort study; (3) limited evidence, one low-quality case-control/cohort studies and/or at least two cross-sectional studies; (4) conflicting evidence, inconsistent findings among multiple studies (case-control/cohort/cross-sectional studies); (5) no evidence, no case-control/cohort/cross-sectional studies reported. 

### 2.6. Data Synthesis and Analysis

The statistical analysis was performed using meta-analyses with interactive explanations (MIX, version 1.7) with the data comparing patients with TMD to asymptomatic participants [27]. 

We employed the same inclusion criteria for the systematic review and the meta-analysis but added two criteria: (1) the Results section contained detailed information on the comparative statistical data (mean, standard deviation, and/or 95% confidence interval) of the main variables and (2) data for the analyzed variables were represented in at least 3 studies. We presented the summary statistics in the form of forest plots [28], which consisted of a weighted compilation of all standardized mean differences (SMDs) and corresponding 95% confidence intervals (CI) reported by each study and provided an indication of heterogeneity among the studies. 

The statistical significance of the pooled SMDs was examined using Hedges’ *g*, to account for possible overestimation of the true population effect size in small studies [29]. The magnitude of *g* was interpreted according to a 4-point scale: (1) <0.20, negligible effect; (2) 0.20–0.49, small effect; (3) 0.50–0.79, moderate effect; (4) ≥0.80, large effect [30]. We estimated the degree of heterogeneity among the studies by employing Cochran’s Q statistic test (*p* < 0.1 was considered significant) and the inconsistency index (I^2^) [31]. I^2^ > 25% is considered to represent low heterogeneity, I^2^ > 50% is considered medium, and I^2^ > 75% is considered to represent large heterogeneity [32]. The I^2^ index is complementary to the Q test, although it has a similar problem of power as the Q test with a small number of studies [32]. Therefore, a study was considered heterogeneous when it fulfilled one or both of these conditions: (1) the Q-test was significant (*p* < 0.1), and (2) the result of I^2^ was >75%. We performed a random-effects model, as described by DerSimonian and Laird [33], in the meta-analysis of the heterogeneous studies to obtain a pooled estimate of effect. To detect publication biases and to test the influence of each study, we performed a visual evaluation of the funnel plot and exclusion sensitivity plot, searching for any asymmetry. We also employed Egger’s regression test to determine the presence of bias [34,35].

## 3. Results

**Figure 1:**
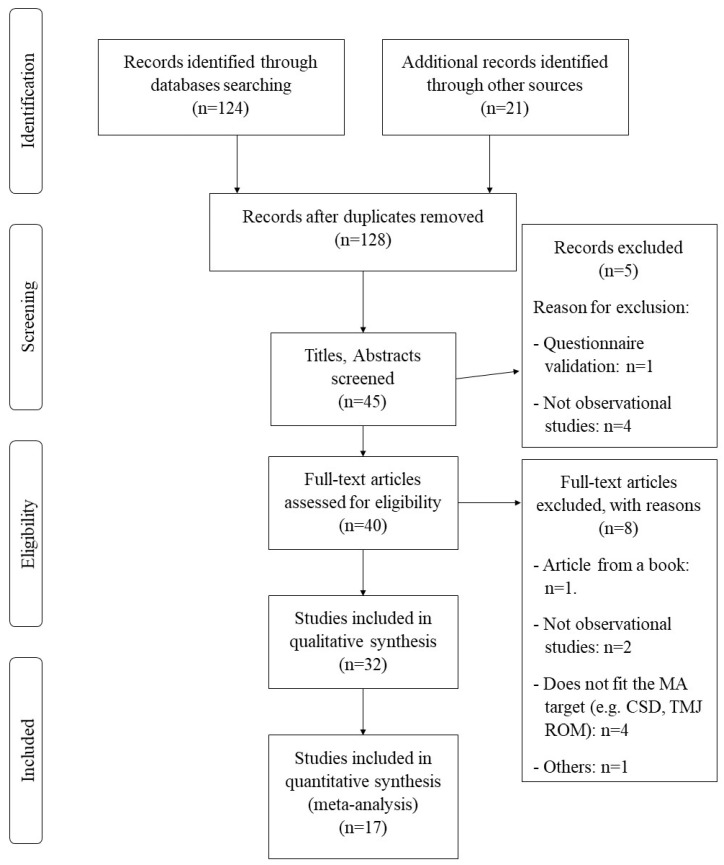
PRISMA flow diagram.

The study search strategy was presented in the form of a flow diagram (Figure 1). A total of 32 articles met the inclusion criteria (three case-control studies and 29 cross-sectional studies) [36,37,38,39,40,41,42,43,44,45,46,47,48,49,50,51,52,53,54,55,56,57,58,59,60,61,62,63,64,65,66,67]. Seventeen articles had been included in three separate meta-analyses. The first meta-analysis included six articles and assessed the correlation between neck disability and the presence of TMD. The second meta-analysis included five articles and dealt with the craniocervical position. The third meta-analysis included six articles and evaluated the position of the head relative to the neck. Table 1 lists the epidemiological characteristics, the results, and the conclusion of each article. 

### 3.1. Temporomandibular (TMD) Diagnosis Criteria 

More than half of the selected studies used the Research Diagnostic Criteria for Temporomandibular Disorders (RDC/TMD) as the preferred diagnostic method [36,37,38,39,40,43,46,47,48,50,51,52,53,54,55,63,67]. The RDC/TMD was first published in 1992 by Dworkin and LeResche, and it provides an assessment of the most common TMD conditions taking into consideration both the clinical condition (Axis I) and the psychosocial status and pain-related disability (Axis II) [16]. Other validated instruments were also used to diagnose TMD: the American Association of Orofacial Pain questionnaire [59,61], Helkimo’s index of mandibular mobility [42], and Conti’s questionnaire of TMD subjective symptoms [48]. On the other hand, four studies carried out a physical exploration in order to give a TMD diagnosis. Finally, in four studies, the samples had already been diagnosed with TMD [45,57,60,64], and in three studies, the TMD diagnosis criteria were not specified [41,58,66].

### 3.2. Results of the Methodological Quality

The agreement between the two evaluators, according to the Kappa coefficient, was high (κ = 0.756). The intervention of a third evaluator was necessary to achieve consensus on the quality of 14 studies.

One case-control study showed a fair methodological quality, with a score of 5 [47]; the other two case-control studies achieved a score of 3 or lower, which is considered poor methodological quality [41,61]. The mean total score for the methodological quality was 3, with a standard deviation of 2.0 and a range of 1–5 points. In most cases, the methodological quality score was affected by the lack of representativeness in the cases. Neither of the case-control studies presented non-response rates for the participants. Seventeen cross-sectional studies showed fair methodological quality, with a score of 2 [37,38,39,40,43,48,50,51,52,53,54,55,57,62,63,66,67]; the other 12 studies achieved a score of 1 or lower, which is considered poor methodological quality [36,42,44,45,46,49,56,58,59,60,64,65]. The mean total score for methodological quality was 1.48, with a standard deviation of 0.68, and a range of 0–2. In most cases, the methodological quality score was affected by the lack of representativeness of the exposed cohort. Table 2 and Table 3 show the numerical results of the NOS scale.

### 3.3. Characteristics of the Study Population

A total of 1903 patients with TMD were included in the experimental group [37,38,39,40,41,42,43,44,45,46,47,48,49,50,51,52,53,54,55,56,58,59,60,61,62,63,64,65,67], and their symptoms lasted at least 3 months. The TMD presented in isolation or concomitantly with other clinical entities, such as neck pain, neck disability, headache, cervicogenic headache, cervical spine disorders, and disc-interference disorders [43,44,46,50,53,54,60,62,63,64]. In terms of the TMD diagnosis, 21 studies contained data on myogenous TMD [36,37,38,39,40,41,43,47,48,50,51,53,55,56,58,60,62,63,64,65,67], 19 studies included data on mixed TMD [36,37,38,39,40,41,43,50,51,52,53,55,56,58,62,63,64,65,67], and nine studies referred to arthrogenous TMD [43,48,50,51,53,58,64,65,67]. Eight studies did not specify the TMD subtype [42,44,45,46,49,54,59,61], and the two remaining studies of this systematic review included patients with cervical-craniofacial pain, craniomandibular disorders, and cervical spine disorders as the experimental group [57,66].

Given that TMD is more frequent in women than in men, the female sex was predominant in most of the studies’ samples. There was only one study in which women comprised the minority [51]. In the rest of the studies, the proportion of women ranged from 55% to 100%, barring one study that did not specify the participants’ sex [41]. The age range defined in the inclusion criteria of the TMD groups was between 18 and 60 years.

### 3.4. Association between Cervical and Mandibular Disability

**Figure 2:**
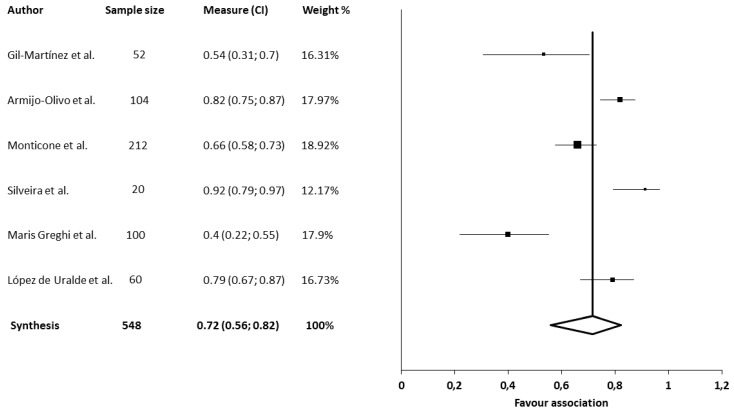
Synthesis forest plot for the association between cervical and mandibular disability. SMD, standardized mean difference. This forest plot summarizes the results of six included studies (sample size, standardized mean differences (SMDs), and weight). The small boxes with the squares represent the point estimate of the effect size and sample size. The lines on either side of the box represent a 95% confidence interval (CI). The horizontal axis represents whether the quantitative analysis is for or against the association.

**Figure A1:**
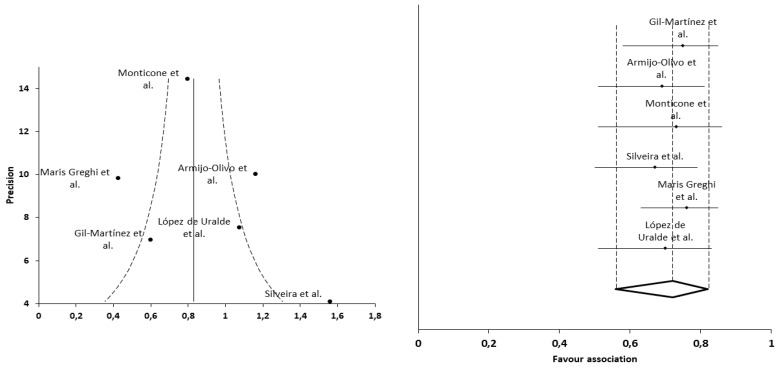
Publication bias heterogeneity funnel plot for the association between cervical and mandibular disability. A funnel plot was used to assess the risk of publication bias. The diagonal lines represent 95% confidence limits.

The association between neck disability and jaw disability in patients with TMD was assessed in six studies [36,51,53,57,58,62]. All of the studies employed the neck disability index for cervical disability. Two of the studies used the jaw function scale for TMJ disability [36,62], and the rest of the studies employed the craniofacial pain and disability inventory [51,53,57,58]. All studies showed significant associations between neck disability and jaw disability in patients with TMD [36,51,53,57,58,62]. The strongest association was found by Silveira et al. [62] (*r* = 0.915), and the lowest association was found by Greghi et al. [53] (*r* = 0.40). The meta-analysis for the association between neck disability and jaw disability in patients with TMD showed statistically significant correlations with a moderate clinical effect (six studies [36,51,53,57,58,62], 548 patients; SMD, 0.72; 95% CI 0.56–0.82) and heterogeneity (Q value, 42.07; *p* < 0.001; I^2^, 88%). The shape of the funnel plot appeared to be symmetrical in the dominant model, as judged by visual examining the intensity of the pain (Figure 2). The influence of each individual study was assessed with a sensitivity exclusion analysis. We obtained statistically strong results because the analysis suggested that no individual study significantly affected the pooled SMD. The similarity found among the pooled estimates suggested that there was no single study influencing the results of the meta-analysis (Annexes; Figure A1).

### 3.5. Craniocervical Position

Craniocervical position was addressed by the following five outcome measures through five studies [48,55,59,61,65]: C0–C1 distance [48,55,61], craniocervical angle [55,61], high cervical angle [48], cranial rotation angle [65], and skull base/odontoid angle [59]. None of the studies showed significant differences. 

**Figure 3:**
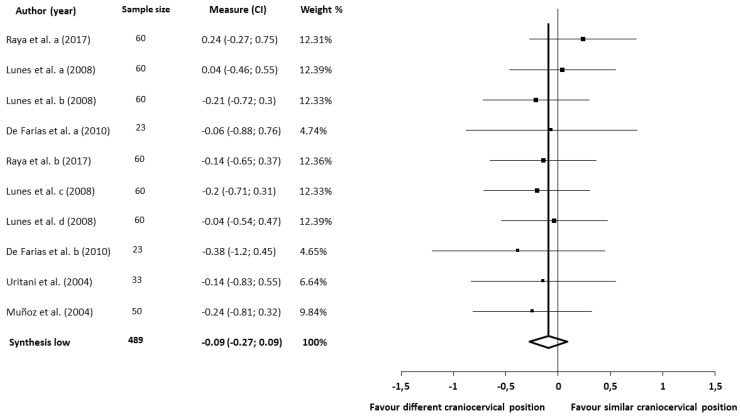
Synthesis forest plot for the craniocervical position. SMD, standardized mean difference. This forest plot summarizes the results of five included studies (sample size, standardized mean differences (SMDs), and weight). The small boxes with the squares represent the point estimate of the effect size and sample size. The lines on either side of the box represent a 95% confidence interval (CI). The horizontal axis represents whether the quantitative analysis is for or against the different craniocervical position in patients with TMD.

**Figure A2:**
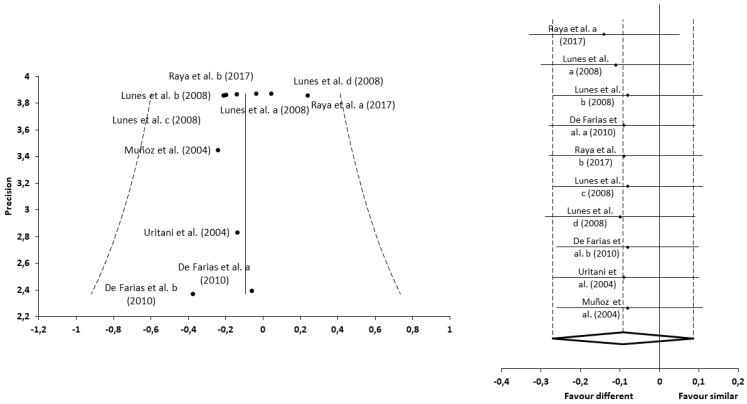
Publication bias heterogeneity funnel plot for the craniocervical position. A funnel plot was used to assess the risk of publication bias. The diagonal lines represent 95% confidence limits.

The meta-analysis for the craniocervical position showed no statistically significant differences (five studies [48,55,59,61,65], 226 patients; SMD, −0.09; 95% CI −0.27 to 0.09) and heterogeneity (Q, 3.12; *p* = 0.96; I^2^, 0%), and there was evidence of publication bias for the meta-analysis (SE, 0.03; T, −7.0; *p* < 0.001). The shape of the funnel plot seemed to be asymmetrical in the dominant model, as judged by visually examining the craniocervical position (Figure 3). The influence of each individual study was assessed with a sensitivity exclusion analysis. We obtained statistically strong results because the analysis suggested that no individual study significantly affected the pooled SMD. The similarity found among the pooled estimates suggested that there was no single study influencing the results of the meta-analysis. Accordingly, we applied Egger’s test of asymmetry, and the results suggested significant evidence of publication bias for the analysis of the craniocervical position (intercept, 2.08; *t*, 6.96; *p* < 0.001) (Figure A2).

### 3.6. Cervical Spine Alignment

Cervical spine alignment was addressed by the following seven outcome measures through six studies [44,48,56,57,65,66]: Tragus-C7 distance over the horizontal plane [44,56,65,66], nasal bridge-C7 [57], cervical posture line (C1–C6) angle over the horizontal plane [66], anterior translation distance (C2–C7 distance) [48], neck-length (from C7 to the tragus)/shoulder-width ratio [65], eye-tragus-C7 angle [56], and the ear-vertical plumb line [56]. 

**Figure 4:**
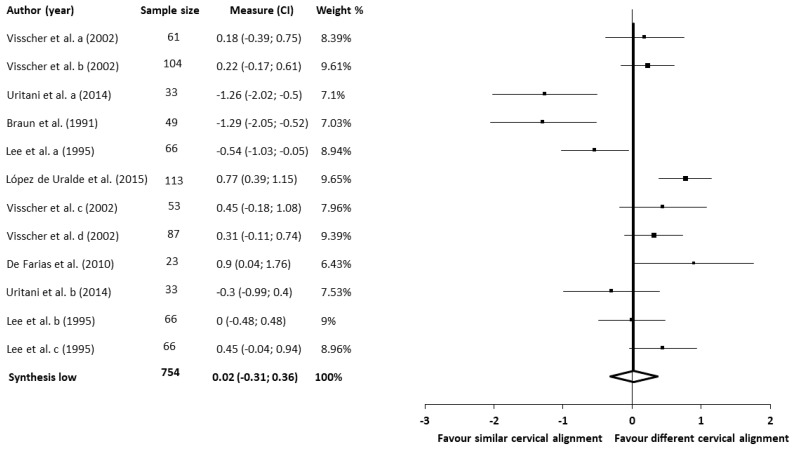
Synthesis forest plot for the cervical spine alignment. SMD, standardized mean difference. This forest plot summarizes the results of six included studies (sample size, standardized mean differences (SMDs), and weight). The small boxes with the squares represent the point estimate of the effect size and sample size. The lines on either side of the box represent a 95% confidence interval (CI). The horizontal axis represents whether the quantitative analysis is for or against the different cervical alignment in patients with TMD.

**Figure A3:**
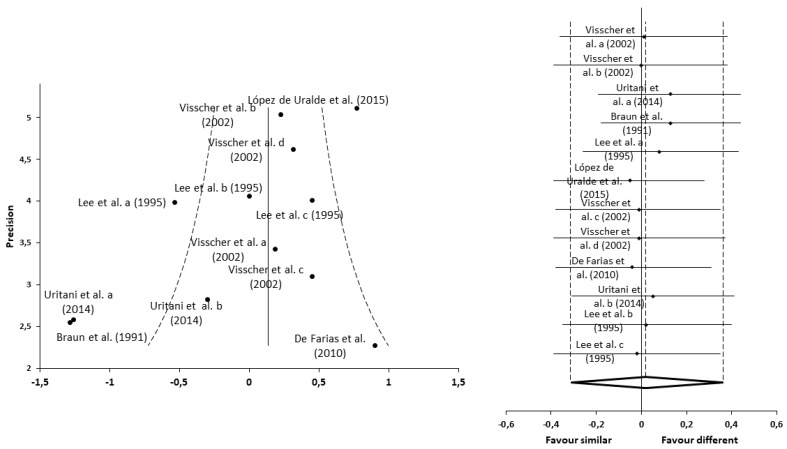
Publication bias heterogeneity funnel plot for the cervical spine alignment. A funnel plot was used to assess the risk of publication bias. The diagonal lines represent 95% confidence limits.

The meta-analysis for the cervical spine alignment showed no statistically significant differences (six studies [44,48,56,57,65,66], 404 patients; SMD, 0.02; 95% CI—0.31–0.36) and heterogeneity (Q, 55.18; *p* < 0.001; I^2^, 79%), and there was no evidence of publication bias for the meta-analysis (SE, 0.03; T, 0.53; *p* = 0.6). The shape of the funnel plot appeared to be asymmetrical in the dominant model as judged by visually examining the position of the head relative to the neck (Figure 4). The influence of each study was assessed with a sensitivity exclusion analysis. We obtained statistically strong results because the analysis suggested that no individual study significantly affected the pooled SMD. The similarity found among the pooled estimates suggested that there was no single study influencing the results of the meta-analysis. Accordingly, we applied Egger’s test of asymmetry, with the results suggesting no significant evidence of publication bias for the analysis of the head position relative to the neck (intercept, 0; *t*,−0.01; *p* = 0.99) (Figure A3).

### 3.7. Pressure Pain Thresholds in the Craniocervical Region

These five studies assessed the mechanosensitivity of masticatory and cervical muscles and orofacial structures using pressure pain thresholds (PPTs) [43,47,62,63,67]. Four of the aforementioned studies employed manual pressure algometers [47,62,63,67], and one employed a digital dynamometer to measure and compare PPTs in patients with TMD and asymptomatic controls [43]. In three studies, the TMD group had concurrent neck disability or neck pain [43,62,63]. PPTs were recorded bilaterally at various anatomical points. In the craniomandibular region, five studies chose the masseter and temporalis muscles [43,47,62,63,67], and one study chose the lateral pole of the TMJ [47]. In the cervical region, four studies used the upper trapezius [47,62,63,67] and sternocleidomastoid muscles [43,47,62,63], two studies used the suboccipital muscles [43,67], and one study used the middle trapezius [43]. The hypothenar and thenar region of the hand and the Achilles tendon were selected as distal points in three studies [43,47,63].

All five studies reached the same conclusion: patients with TMD, regardless of the presence of neck disability or neck pain, showed significantly lower PPTs at almost all craniocervical structures when compared with the control group [43,47,62,63,67]. Altogether, there was moderate evidence of lower PPTs in patients with TMD.

### 3.8. Cervical Spine Range of Motion

Seven studies analyzed the cervical range of movement (ROM) in patients with TMD [42,45,46,50,54,64,67]. Three of these studies compared ROM between patients with TMD and asymptomatic controls using a cervical ROM (CROM) instrument [50], the Keno^®^-cervical measurement instrument [67], and through visual evaluation [45]. Three other studies evaluated active ROM using a goniometer [42,46,67] and an inclinometer [54] on patients with TMD and those with TMD and concurrent disorders. The remaining study used CROM to measure the ROM of patients with TMD and concurrent neck or headache disorders and patients with headache or neck pain [64]. All of the aforementioned studies assessed flexion, extension, and both lateral flexions and rotations in the cervical spine. 

In four studies, the patients with TMD presented significant limitations in flexion, extension [50,54,67], and both lateral flexion movements [46] compared with the asymptomatic participants. In the remaining three studies, concise conclusions could not be drawn due to various reasons: the results could only be expressed as correlations [64] were nonexistent [45] or were expressed in percentages and related to TMD severity [42].

Furthermore, three studies included the flexion-rotation test as a measurement of ROM [50,54,67]. Two of the studies showed that both rotation movements were significantly lower in patients with TMD compared with the control group [50,54], and one study found no relevant differences between the groups [67]. In summary, there was moderate evidence of limited cervical ROM in patients with TMD.

### 3.9. Cervical Strength

Five studies observed the cervical strength of patients with TMD compared with control participants [37,38,39,41,67].

Two studies analyzed the maximal cervical flexor strength using a cervical flexor strength device that monitored the force generated by the participants with a load cell [37,41]. In both studies, there was no significant difference in maximal cervical flexor strength between the patients with TMD and the asymptomatic participants [37,41].

Four studies evaluated the cervical endurance of patients with TMD compared with the control group [38,39,41,67]. Three studies measured the cervical flexor endurance [38,41,67], and two studies measured the cervical extensor endurance [39,41]. One study found no significant differences in cervical flexor endurance between the patients with TMD and the control group [67]. However, two studies found significant differences in holding time when the cervical flexor endurance test was performed at 25% of maximal voluntary contraction, with less holding time in the TMD group than in the control group [38,41]. Cervical extensor endurance was significantly lower in the TMD group than in the control group [39,41].

Consequently, there was limited evidence for equal values in maximal cervical flexor strength between the patients with TMD and the control group. There was also limited evidence of reduced cervical endurance in patients with TMD.

### 3.10. Electromyographic Activity in Cervical Muscles

Four studies compared the electromyographic (EMG) activity in the neck muscles of patients with TMD and control participants [39,40,41,60]. Three studies compared EMG activity in the superficial neck muscles [40,41,60], and one study compared the EMG activity in the neck extensor muscles [39]. Specifically, one study measured EMG activity in the sternocleidomastoid and trapezius muscles at rest [60], two studies measured EMG activity in the sternocleidomastoid and anterior scalene muscles while performing the craneo-cervical flexor test (CCFT) [40,41], and the remaining study measured EMG activity in the extensor muscles while performing the neck extensor muscle endurance test (NEMET) [39].

There were no significant differences in EMG activity in the sternocleidomastoid and anterior scalene muscles in the patients with TMD when compared with the asymptomatic participants [40,41]. However, the patients with TMD had a significantly higher resting EMG activity in the sternocleidomastoid and trapezius muscles when compared with the asymptomatic participants [60]. There were significant differences in EMG activity during the NEMET, which showed higher fatigability of the cervical extensor muscles in the patients with TMD [39]. As a result, there was conflicting evidence regarding abnormal EMG activity in patients with TMD.

### 3.11. Cervical Motor Control

Three studies evaluated the motor control of cervical flexors in patients with TMD using the CCFT [40,50,67]. Two studies found no significant differences in CCFT performance between the patients with TMD and the control group [40,67]. Meanwhile, one study showed significantly lower pressures during the CCFT performance (a finding related to poorer motor control) in the TMD group than in the control group [50]. As a consequence, conflicting evidence regarding abnormal cervical motor control was shown in patients with TMD.

## 4. Discussion

The aim of this systematic review and meta-analysis was to assess whether cervical and mandibular disabilities were related in patients with TMD and to determine the possible differences in craniocervical posture, cervical spine alignment, and cervical sensory-motor function in these patients compared with asymptomatic participants. Several studies have reported an association between cervical pain and TMD [36,39,43,47,68,69], which might be explained by the neuroanatomical link between the orofacial and cervical regions [15,69,70]. However, disability is a complex concept influenced by the patient’s perception of their condition [36,62]; some patients with severe TMD have low levels of disability and low impact on their quality of life [36,62]. Therefore, the degree of disability depends only partly on the patient’s signs and symptoms [38,40,50,62,64]. We, therefore, considered this systematic review relevant because it was the first to analyze the relationship between the two regions in terms of disability. The results revealed that patients with TMD presented jaw disability moderately related to their degree of cervical disability. These patients also presented sensory-motor impairments (but not postural) in the cervical region compared with the asymptomatic participants. 

The association between TMD and cervical disorders has been an area of interest for many years, a relationship attributed to the neurophysiological, biomechanical, and functional link between the two regions [15,68,70,71,72]. Our results suggested that the neurophysiological component might be more important than the biomechanical in explaining the observed disorders. For example, the study conducted by Favia et al. [73] showed the role of neuroreceptors in TMD. This hypothesis is reinforced by the lack of differences in craniocervical posture and cervical alignment between patients with TMD and asymptomatic participants. Furthermore, the reported quantitative analysis provides more evidence than that of two previous systematic reviews on the subject, which showed inconclusive results [15,74]. However, we considered that the relevance of posture in these patients should not be completely ruled out because their assessment could be influenced by the Hawthorne effect [75]. Patients might, therefore, not adopt their actual posture when asked to position themselves in a specific manner in preparation for radiography [48,55,59,61,66]. In fact, patient monitoring over a temporary period, and not just momentarily, seems to be a determining factor in identifying postural alterations [76]. These factors should be considered in future studies to establish more conclusive results.

The impact of mandibular disability on perceived neck disability is evident, with the results reflecting a relationship in the clinical impact due to the resulting size of the effect (moderate/large with a *g* of 0.72). However, the mechanisms underlying this relationship are currently unknown [62]. The best explanation might be the neurophysiological connection between the two regions of the trigeminocervical nucleus [70,72,77,78]. Painful afferences from the temporomandibular region would, therefore, sensitize the cervical region [79,80]. A number of articles in the literature support this hypothesis, showing an association between pain intensity and perceived disability [18,50,81,82,83]. In contrast, however, it could be argued that disability is a phenomenon not entirely explained by pain intensity, with numerous other relevant aspects, such as psychosocial factors [18,84,85]. However, most of the analyzed studies included patients with chronic TMD, thereby showing a certain predisposition to central sensitization [86,87], as well as to cognitive/emotional maladaptive factors [88,89,90]. It is, therefore, possible that the relevance of nociceptive information gains greater prominence in explaining the relationship in disability between the orofacial and cervical regions.

Regarding sensory-motor disorders, the most conclusive findings were observed in the reduction of PPT and cervical ROM, with moderate evidence of this reduction in patients with TMD. These disorders could be due to an increase in cervical muscle activity, which a number of authors have attributed to changes in head and neck position [91,92]. However, the lack of differences at the postural level gives greater plausibility to the neurophysiological hypothesis than to the biomechanical one. Thus, the reduction in PPT could be attributed to ischemia caused by sustained contraction [93,94], which could also explain the reduction in ROM by changes in cervical neuromuscular control (e.g., co-contraction of antagonistic agonists, increased co-activation of synergistic muscles, and/or increased activity of superficial muscles at rest) for protective purposes [95,96,97,98]. Along the same lines, a number of authors have reported the so-called trigeminocervical reflex as a possible physiopathological mechanism [99], a phenomenon that demonstrates the effect of mechanoreceptors and nociceptors of TMJ on the fusimotor-muscular spindle system of the cervical muscles [100,101,102]. Abnormalities in cervical neuromuscular control could, therefore, be the result of an overload of the cervical structures due to increased muscle activity. According to the Cinderella hypothesis [103], long-lasting muscular activity and low-intensity loading can activate small type-I motor units in a selective and continuous manner [104,105,106]. The metabolic disorders produced by this event would, therefore, result in tissue damage and, most likely, pain [107,108].

However, the evidence regarding the impairment of motor control and cervical EMG activity in patients with TMD is conflicting. The number of studies was limited, as were some of the aspects from studies that established the lack of differences between these patients and asymptomatic participants. Specifically, the results of the studies conducted by Armijo-Olivo et al. [40,41] showed that the magnitude of the difference was clinically relevant, despite showing no statistically significant differences. Therefore, the lack of differences could be due to a type II error as a consequence of EMG measurements having high variability [40]. In contrast to most of the studies included in this review that studied patients with chronic TMD, the study by von Piekartz et al. [67] was conducted with acute/subacute patients with an average pain intensity of fewer than three points on the VAS. Therefore, the low intensity and duration of their symptoms might be insufficient to sensitize the trigeminocervical nucleus and cause disorders in the cervical region. Future studies that consider these aspects should, therefore, provide definite conclusions on the presence of disorders in motor control and EMG activity in patients with TMD.

### 4.1. Clinical Implications

Clinically, these results suggest that patients with TMD show sensorimotor but not postural impairments in the cervical region compared with asymptomatic participants. Although these results should be interpreted with caution due to the methodological quality of the included studies, the results could help increase clinicians’ understanding of the effect of TMD in these patients and thereby help apply the optimal treatment. 

A recent review by Gil-Martínez et al. [51] reported that neck disability was a strong predictor of craniofacial pain and disability in a subgroup of patients with TMD due to muscle pain and that neck disability had a positive correlation with orofacial pain and disability, kinesiophobia, and pain catastrophizing. These findings suggest the possibility of including a new therapeutic approach for patients with TMD. Based on our results, future interventions applied to patients with TMD should address their psychosocial behavior to improve the cervical and mandibular disability observed in these patients. However, there is limited evidence on the efficacy of an approach based on psychosocial factors in improving disability in patients with TMD, and future clinical trials addressing this issue are needed. 

### 4.2. Limitations

This review presents a number of limitations. First, the design of the studies prevented a cause-effect relationship from being established. Future studies using cohort design and especially experimental studies are needed to better understand how TMD influence neck disorders. Secondly, the methodological quality of the studies was fair/poor, and, therefore, the results should be interpreted with caution. We could not perform quantitative analysis for the neck sensorimotor variables or a comparison between the various TMD diagnoses (mixed, myogenic, and arthrogenous) due to the scarcity of studies. Based on their possible influence on the results, future studies need to consider these aspects to establish more conclusive results. Finally, the meta-analysis for the craniocervical position showed significant evidence of publication bias, which should also be taken into account. 

## 5. Conclusions

The results of this study showed a clinically relevant association between cervical and mandibular disability in patients with TMD. These patients also showed sensorimotor but not postural impairments in the cervical region compared with the asymptomatic participants. Specifically, patients with TMD experienced reduced PPT and cervical ROM (moderate evidence) and loss of cervical muscle endurance (limited evidence). However, maximal cervical musculature strength was not changed (limited evidence). Finally, there was conflicting evidence regarding the impairment of EMG activity and cervical motor control in patients with TMD. 

## Figures and Tables

**Table 1:** Characteristics of included studies.

Article Design	Sample Characteristics	Inclusion Criteria in the Experimental Group	Outcomes Measures	Results
Da Costa et al., 2015 Case-control	**Experimental group**	18–35 years, pain in the orofacial region, masticatory myofascial pain diagnosis according to RDC/TMD	**Neck disability** Self-reported using NDI **Sensory-motor impairments** PPT in masticatory structures, cervical muscles, and the extracephalic site using a digital dynamometer	**Neck disability** TMD patients showed greater neck disability compared to the asymptomatic group **Sensory-motor impairments** TMD patients had lower PPTs values compared to the asymptomatic group
	Myogenous TMD patients (≥6-months) *N* = 27 (22 F/5 M) Age: 24.7 ± 3.7 years			
	**Control group**			
	Healthy subjects; *N* = 28 (17 F/11M) Age: 23.2 ± 3.8 years			
Raya et al., 2017 Case-control	**Experimental group**	18–30 years, TMD symptoms using the AAOP questionnaire	**Craniocervical position** C0–C1 distance, craniocervical angle using X-ray examination	**Craniocervical position** Women with and without TMD showed similar C1–C0 distances and craniocervical angles. Alterations in the craniocervical position were not correlated with TMD symptomatology
	TMD patients (2 episodes ≥ 12-months); *N* = 30 (F) Age: 24.2 ± 3.1 years			
	**Control group**			
	Healthy subjects; N = 30 (F) Age: 23.5 ± 2.9 years			
Armijo-Olivo et al., 2011 Case-control	**Experimental group**	Undescribed	**Sensory-motor impairments** Maximal cervical flexor strength and endurance of the cervical flexor using visual feedback device, extensor muscles using NEMET and stopwatch and EMG activity of the cervical flexor muscles	**Sensory-motor impairments** There were no statistically significant differences between the TMD groups and the control group related to the EMG activity of the cervical flexor muscles and maximal cervical flexor strength. Both TMD groups showed lower holding time when evaluating the endurance of the cervical flexor and extensor muscles
	Myogenous TMD patients; *N* = 56 Mixed TMD patients; *N* = 48			
	**Control group**			
	*N* = 50			
Gil-Martínez et al., 2017 Cross-sectional	**Experimental group**	≥18 years, mixed chronic orofacial pain according to RDC/TMD	**Disability** Self-reported, neck disability using NDI, and craniofacial disability using CF-PDI	**Disability** The mixed chronic TMD patients showed significantly higher scores in craniofacial disability and similar scores in neck disability compared to chronic migraine patients
	Mixed chronic TMD patients (106.1 ± 62.2 months); *N* = 51 (40 F/11 M) Age: 46.2 ± 11.7 years			
	**Control group**			
	Chronic migraine patients; *N* = 50 (46 F/4 M) Age: 48.6 ± 13.2 years			
Thorp et al., 2019 Cross-sectional	**Experimental group**	≥18 years, no history of neck or TMJ surgery, a medical diagnosis of cervicogenic headache, mechanical neck pain, and/or TMD	**Neck disability** Self-reported using NDI **Sensory-motor impairments** Cervical AROM using CROM ^TM^ device	**Neck disability** No statistical difference in neck disability score was identified among the three groups. The TMD patients with neck pain and cervicogenic headache showed the lowest score in neck disability compared to the other two groups; however, NDI scores were not correlated with TMD pain **Sensory-motor impairments** Significant inverse correlations of the neck disability with all cervical AROM across all participants
	Myogenous, arthrogenous, or mixed TMD with neck pain and cervicogenic headache patients (71.3 ± 50.9 months); *N* = 15 Age: 39.5 ± 17.5 years			
	**Control group**			
	Neck pain patients; *N* = 17 Age: 57.5 ± 9.6 years Neck pain and cervicogenic headache patients: *N* = 30 Age: 50.6 ± 17.5 years			
Gil-Martínez et al., 2016 Cross-sectional	**Experimental group**	Medical diagnosed chronic painful TMD according to RDC/TMD	**Disability** Self-reported craniofacial disability using CF-PDI and neck disability using NDI	**Disability** The mixed TMD patients showed greater craniofacial and neck disability compared to the other groups of patients. The arthrogenous patients showed greater neck disability than myogenous patients. The myogenous and the mixed TMD patients showed a moderate positive correlation between neck disability and craniofacial disability
	*N* = 154 (66 F/88 M) Age: 45.2 ± 12.8 years Chronic arthrogenous TMD patients; *N* =43 (24 F/19M) Chronic myogenous TMD patients; *N* = 59 (25 F/34 M) Chronic mixed TMD patients; *N* = 52 (17 F/35M)			
	**Control group**			
	No control group			
Silveira et al., 2015 Cross-sectional	**Experimental group**	Diagnosed TMD according to RDC/TMD and presented concurrent neck disability	**Disability** Self-reported neck disability using NDI, jaw disability using LDF-TMDQ **Sensory-motor impairments** PPT in masticatory and cervical muscles using a manual pressure algometer	**Disability** The jaw disability and neck disability were strongly correlated **Sensory-motor impairments** Subjects with TMD, regardless of the presence of neck disability or neck pain, showed significantly lower PPTs at almost all craniocervical structures when compared with subjects of the control group
	Myogenous or mixed TMD patients (≥3 months); *N* = 20 (F) Age: 31.1 ± 6.9 years			
	**Control group**			
	Healthy subjects; *N* = 20 (F) Age: 32.3 ± 7.2 years			
Bragatto et al., 2016 Cross-sectional	**Experimental group**	20–50 years, working at the same job for at least 12 months, computer use for ≥4 h/day at work and diagnosed TMD according to RDC/TMD	**Neck disability** Self-reported using NDI **Sensory-motor impairments** Mechanical pain was assessed by manual palpation and PPTs in masticatory and cervical muscles using a digital dynamometer	**Neck disability** A neck disability was influenced by TMD and neck pain **Sensory-motor impairments** PPT of craniocervical structures was significantly lower among computer workers regardless of neck pain
	Myogenous, arthrogenous, or mixed TMD computer workers; *N* = 26 (F) Age: 33.8 years Myogenous, arthrogenous, or mixed TMD with concurrent neck pain computer workers (≥3 months); *N* = 26 (F) Age: 36.5 years			
	**Control group**			
	Asymptomatic non-computer workers; *N* = 26 (F) Age: 26.2 years			
Coskun et al., 2018 Cross-sectional	**Experimental group**	Diagnosis of TMD according to RDC/TMD	**Sensory-motor impairments** Cervical AROM using goniometer	**Sensory-motor impairments** The TMD with concurrent neck pain group showed significant lower ROM in both side flexions compared to the TMD group
	TMD patients; *N* = 32 (26 F/6 M) Age: 30.1 ± 11.4 years TMD with concurrent neck pain patients: *N* = 28 (24 F/4 M) Age: 32.5 ± 10.2 years			
	**Control group**			
	No control group			
Greghi et al., 2018 Cross-sectional	**Experimental group**	Diagnosis of painful TMD according to RDC/TMD, a history of orofacial pain, headaches, and neck pain, no cognitive deficits	**Disability** Self-reported craniofacial disability using CF-PDI, neck disability using NDI, orofacial disability using MFIQ, and pain-related disability using PDQ	**Disability** Significative association between neck, orofacial, and pain-related disability with respect to craniofacial disability in patients with TMD was found
	Myogenous, arthrogenous, or mixed TMD patients with and without other orofacial pains (≥6 months); *N* = 100 (89 F/11 M) Age: 39.8 ± 16.2 years			
	**Control group**			
	No control group			
Monticone et al., 2019 Cross-sectional	**Experimental group**	Adult age, headache, or facial pain attributable to TMD due to untreated muscular, articular, or mixed complaints and a chronic condition defined as pain history	**Disability** Self-reported, craniofacial disability using CF-PDI, and neck disability using NDI	**Disability** Correlation analyses showed that TMD was closely associated with neck disability
	Myogenous, arthrogenous, or mixed TMD patients (≥12 months); *N* = 212 (177 F/35 M) Age: 47.7 ± 14.2 years			
	**Control group**			
	No control group			
López de Uralde-Villanueva et al., 2015 Cross-sectional	**Experimental group**	18–65 years, diagnosis of chronic cervico-craniofacial pain of muscular origin, disability, and pain in these regions according to the CF-PDI, diagnosis of myofascial pain according to RDC/TMD and bilateral pain of the masticatory and cervical muscles	**Disability** Self-reported, craniofacial disability using CF-PDI, and neck disability using NDI **Cervical spine alignment** Head posture using the CROM^TM^ device. The sternomental distance using plastic digital caliper with a five-digit LCD display	**Disability** There was no association between craniocervical posture and pain-related disability A strong correlation between the neck and craniofacial disability was found **Cervical spine alignment** A moderate positive correlation was observed between craniocervical posture variables for both groups
	Chronic cervico-craniofacial pain patients (≥6 months); *N* = 60 (32 F/28 M) Age: 41.7 ± 11.7 years			
	**Control group**			
	Healthy subjects; *N* = 53 (30 F/23 M) Age: 38.1 ± 10.5 years			
Armijo-Olivo et al., 2010 Cross-sectional	**Experimental group**	18–50 years, moderate or severe pain in the masticatory muscles/temporomandibular joint not attributable to recent acute trauma, active inflammatory cause, or previous infection. Diagnosis of myogenous TMD according to Dworkin and LeResche classification	**Disability** Self-reported, neck disability using NDI, jaw disability using LDF-TMDQ⁄JFS, and level of chronic TMD disability using RDC/TMD	**Disability** There was a strong association between neck disability and jaw disability. The jaw disability was significantly higher for patients with mixed TMD compared to myogenous TMD patients
	Myogenous TMD patients (6.5 ± 6.3 years); *N* = 56 (F) Age: 31.1 ± 8.9 years Mixed TMD patients (8.2 ± 6.4 years); *N* = 48 (F) Age: 31.5 ± 8.2 years			
	**Control group**			
	Healthy subjects *N* = 50 (F) Age: 28.3 ± 7.3 years			
Silveira et al., 2014 Cross-sectional	**Experimental group**	18–50 years, TMD diagnosed according to RDC/TMD, chronic orofacial pain not attributed to recent acute trauma, previous infection, or an inflammatory cause	**Disability** Self-reported, neck disability using NDI, and jaw disability using JDI **Sensory-motor impairments** PPT in masticatory structures, cervical muscles, and the extracephalic site using a manual pressure algometer	**Disability** The jaw disability was significantly higher than neck disability in patients with TMD **Sensory-motor impairments** There was a significant increase in the tenderness of the masticatory and cervical muscles in the TMD patients compared to the healthy subjects
	Myogenous or mixed with concurrent neck disability patients (≥ 3 months); *N* = 20 (F) Age: 31.1 ± 6.9 years			
	**Control group**			
	Healthy subjects; *N* = 20 (F) Age: 32.3 ± 7.2 years			
Visscher et al., 2002 Cross-sectional	**Experimental group**	Diagnosis of chronic musculoskeletal disorders, such as a painful CMD or CSD	**Cervical spine alignment** Head posture using lateral photographs and lateral X-ray examination of the head and cervical spine	**Cervical spine alignment** No difference was found related to head posture between the CMD with and without CSD patients, the CSD patients, and the healthy subjects. For the photographs, increasing age was associated with a more anteroposition of the head
	CMD patients (≥3 months); *N* = 16 CSD patients (≥3 months); *N* = 10 CMD and CSD patients (≥3 months); *N* = 65 Myogenous CDM patients; *N* = 82 Arthrogenous CDM patients; *N* = 14 Mixed CDM patients; *N* = 15			
	**Control group**			
	Healthy subjects; *N* = 47			
Armijo-Olivo et al. 2010 b Cross-sectional	**Experimental group**	Pain in the masticatory muscles/TMJ of at least 3 months, moderate or severe baseline pain score of ≥30 mm using a 100 mm VAS	**Sensory-motor impairments** Cervical flexion force and endurance using a cervical flexion force device and stopwatch	**Sensory-motor impairments** The mixed TMD group had less endurance capacity at a lower level of contraction compared to the myogenous TMD group and the control group
	Myogenous TMD patients (6.53 ± 6.6 years) *N* =54 (F) Age: 31.63 ± 9.15 years Mixed TMD patients (8.01 ± 6.36 years) *N* = 46 (F) Age: 31.02 ± 8.04 years			
	**Control group**			
	*N* = 49 (F) Age: 28.35 ± 7.32 years			
Armijo- Olivo et al., 2012 Cross-sectional	**Experimental group**	18–50 years, pain in the masticatory muscles/TMJ of at least 3 months not attributable to recent acute trauma, active inflammatory cause, or previous infection, a moderate or severe baseline pain score of ≥30 mm using a 100 mm VAS	**Disability** Self-reported, neck disability using NDI, jaw disability using JFS, and level of chronic TMD disability using RDC/TMD **Cervical spine alignment** Head and neck posture was measured using a lateral photograph **Sensory-motor impairments** Strength and endurance of the cervical muscles using a visual feedback screen through the evaluation of the holding time and EMG activity during NEMET and CCFT	**Disability** A strong association between neck disability and jaw disability was found **Cervical spine alignment** Craniocervical posture was significantly different between patients with myogenous TMD compared to healthy subjects **Sensory-motor impairments** In cervical flexor muscles, there were no significant differences in maximal isometric, nor in EMG activity in patients with TMD compared to healthy subjects; however, mixed TMD patients had less endurance capacity at a lower level of contraction than myogenous TMD patients and healthy subjects. Endurance of cervical extensor muscles was significantly reduced in TMD patients compared to the control group
	Myogenous TMD patients (6.22 ± 6.33 years); *N* = 57 (F) Age: 31.11 ± 8.70 years Mixed TMD patients (8.22 ± 6.50 years); *N* = 47 (F) Age: 31.38 ± 8.42 years			
	**Control group**			
	Healthy subjects *N* = 47 (F) Age: 28.26 ± 7.46 years			
De Laat et al., 1998 Cross-sectional	**Experimental group**	A subjective and untreated complaint of the masticatory system, no past evaluations or treatments for cervical problems	**Sensory-motor impairments** Cervical ROM using a plastic ruler	**Sensory-motor impairments** The TMD group presented greater segmental limitations in the C0–C1 and C2–C3 levels of the cervical spine
	*N* = 31 (24 F/7 M) Age: 36.4 ± 13.5 years			
	**Control group**			
	*N* = 30 (23 F/7 M) Age: 32.3 ± 13.7 years			
Ferreira et al., 2019 Cross-sectional	**Experimental group**	TMD diagnosis, as determined by RDC/TMD, moderate to severe pain in the temporomandibular region lasting for at least 3 months	**Sensory-motor impairments** AROM and PROM of C1–C2 using CROM^TM^ and FRT **Sensory-motor impairments** Performance of the deep cervical flexors using CCFT	**Sensory-motor impairments** Women with TMD and with or without self-reported headaches showed limited flexion and extension ROM, limited C1–C2 mobility **Sensory-motor impairments** Women with TMD and with or without self-reported headaches showed poor deep cervical flexor performance
	Myogenous, arthrogenous, or mixed TMD patients (≥3 months); *N* = 15 Age: 40.33 ± 10.70 years Myogenous, arthrogenous, or mixed TMD with concurrent headache patients (≥3 months); *N* = 25 Age: 35.80 ± 10.04 years			
	**Control group**			
	*N* = 17 (F) Age: 35.64 ± 11.64 years			
Grondin et al., 2015 Cross-sectional	**Experimental group**	Female gender, 18–60 years, a history of side dominant TMD pain for at least 3 months, diagnosis of TMD based on the classification of Dworkin and LeResche, pain score of 30 mm on a 100 mm VAS at rest or during mouth opening	**Sensory-motor impairments** Cervical spine flexion and extension AROM using inclinometer and rotation PROM using FRT and CROM^TM^	**Sensory-motor impairments** All subjects in the TMD group presented ROM restriction compared to those in the control group. Subjects with TMD had signs of impaired movement in the upper cervical spine, which was higher in those with a headache. The TMD group with a headache had less axial rotation than the TMD group without a headache. Only subjects with both TMD and headache had impaired mobility of the sagittal plane of the cervical spine
	TDM with or without headache patients (25.6 ± 32.8 months) *N* = 37 (F) Age: 34.68 ± 12 years			
	**Control group**			
	*N* = 20 (F) Age: 30.6 ± 7.3 years			
von Piekartz et al., 2016 Cross-sectional	**Experimental group**	TMD diagnosis, as determined by the RDC/TMD	**Sensory-motor impairments** PPT in masticatory structures, using a manual pressure algometer and endurance and synergy of the deep cervical flexors using CCFT and pressure stabilizer biofeedback device **Sensory-motor impairments** Cervical AROM and PROM of C1–C2 using Keno^®^-cervical, FRT, and digital goniometer	**Sensory-motor impairments** A higher presence of cervical impairments was found in people with more severe levels of TMD. People with mild and moderate TMD reported lower mechanosensitivity over upper trapezius and obliquus capitis inferior muscles compared to the control group **Sensory-motor impairments** In contrast, the FRT and the CCFT were not impaired in people with TMD
	Mild arthrogenous, myogenous, or mixed TMD patients; *N* = 59 (18 F/41 M) Age: 33.21 ± 10.8 years Moderate/severe arthrogenous, myogenous, or mixed TMD patients; *N* = 40 (34 F/6 M) Age: 37.25 ± 13.78 years			
	**Control group**			
	*N* = 45 (30 F/15 M) Age: 33 ± 8.71 years			
Iunes et al., 2009 Cross-sectional	**Experimental group**	TMD diagnosed by the RDC/TMD and the Fonseca anamnesis index	**Cervical spine alignment** C0–C1 distance and craniovertebral angle using radiographs and correlometer	**Cervical spine alignment** The results of the radiographs revealed that head and cervical spine posture did not differ between both TMD groups and the control group
	Myogenous TMD patients; *N* = 30 (F) Age: 29.13 ± 11.45 years Mixed TMD patients; *N* = 30 (F) Age: 28.13 ± 9.42 years			
	**Control group**			
	*N* = 30 (F) Age: 26.17 ± 9.18 years			
Armijo-Olivo et al., 2010 c Cross-sectional	**Experimental group**	18–50 years, pain in the masticatory muscles/TMJ of at least 3 months not attributable to recent acute trauma, active inflammatory cause, or previous infection, moderate or severe baseline pain score of ≥30mm using a 100-mm VAS	**Sensory-motor impairments** Maximal cervical flexor muscle strength using cervical flexion strength device	**Sensory-motor impairments** Maximal strength of the cervical flexor muscles did not show significant differences among patients with mixed and myogenous TMD and asymptomatic subjects
	Myogenous TMD patients (≥3 months); *N* = 54 (F) Age: 31.63 ± 9.15 years Mixed TMD patients (≥3 months); *N* = 45 (F) Age: 31.07 ± 8.12 years			
	**Control group**			
	*N* = 50 (F) Age: 28.28 ± 7.26			
Armijo-Olivo et al., 2011 b Cross-sectional	**Experimental group**	Female gender, 18–50 years, pain in the masticatory muscles or TMJ of at least 3 months, moderate or severe baseline pain score of ≥30 mm on a 100-mm VAS	**Sensory-motor impairments** EMG activity and performance of the flexor cervical muscles using CCFT and a pressure biofeedback unit	**Sensory-motor impairments** There were no statistically significant differences in EMG activity in the sternocleidomastoid and anterior scalene muscles during the CCFT in the mixed and myogenous TMD groups compared with the control group. However, those with TMD tended to have an increased activity of the superficial cervical muscles compared with the control group
	Myogenous TMD patients (6.5 ± 6.4 years); *N* = 54 (F) Age: 31.4 ± 9 years Mixed TMD patients (8.3 ± 6.4 years); *N* = 49 (F) Age: 31.3 ± 8.3 years			
	**Control group**			
	*N* = 47 (F) Age: 28.3 ± 7.5 years			
Bevilaqua-Grossi et al., 2007 Cross-sectional	**Experimental group**	Female gender, clinical signs and symptoms of TMD and CSD according to clinical indices of Helkimo, Wallace, and Klineberg, respectively	**Sensory-motor impairments** Cervical mobility using ICM	**Sensory-motor impairments** Differences in the values of cervical ROM among TMD severity groups were not confirmed
	TMD and CSD patients; *N* = 100 (F) Age: 21.43 ± 1.8 years			
	**Control group**			
	No control group			
Clark et al., 1987 Cross-sectional	**Experimental group**	Presence of TMD, lack of previous treatment for a craniocervical problem, desire to participate in the study	**Sensory-motor impairments** Cervical ROM using visual examination	**Sensory-motor impairments** There were no significant differences between groups referred to cervical ROM
	*N* = 40 (37 F/3 M) Age: 33.9 ± 12.7 years			
	**Control group**			
	*N* = 40 (37 F/3 M) Age: 33.5 ± 6.8 years			
De Farias et al., 2010 Cross-sectional	**Experimental group**	18–30 years, subjective symptoms of TMD, TMD diagnosis determined by RDC/TMD	**Cervical spine alignment** C0–C1 distance, HCA, and anterior translation distance using radiographs	**Cervical spine alignment** The anterior translation distance showed statistical differences between the TMD group and the control group. No statistical differences were found between the TMD group and the control group for HCA and C0–C1 distance
	Myogenous or arthrogenous TMD patients; *N* = 12 (7 F/5 M) Age: 22.5 ± 4 years			
	**Control group**			
	*N* = 11 (7 F/4 M) Age: 20 ± 2.5 years			
Uritani et al., 2014 Cross-sectional	**Experimental group**	Female gender, 20–49 years, no history of surgery on the upper quadrant, the absence of mental illness or its possibility, and diagnosis of TMD based on myalgia of the masticatory muscle and/or TMJ disc derangement	**Cervical spine alignment** Cranial rotation angle, head posture (tragus-C7-horizontal plane), and neck-length/shoulder-width ratio using 3D motion analyzer	**Cervical spine alignment** No significant differences were found in the outcome measures between the two groups
	Myogenous, arthrogenous, or mixed TMD patients; *N* = 19 (F) Age: 30.1 ± 8.9 years			
	**Control group**			
	*N* = 14 (F) Age: 24.6 ± 6.1 years			
Munhoz et al., 2004 Cross-sectional	**Experimental group**	TMD diagnosis determined by the classification of TMJ ID symptoms of the AAOP	**Cervical spine alignment** Cranium base/odontoid apophysis angle using radiographs	**Cervical spine alignment** No significant differences were found among TMD subgroups and asymptomatic group in the cranium base/odontoid apophysis angle
	*N* = 30 (27 F/3 M) Age: 22.9 ± 5.31 years Mild severity patients (62.8 ± 58.74 months); *N* = 15 Age: 22.4 ± 5.85 years Moderate severity patients (96 ± 84.85 months); *N* = 9 Age: 22 ± 3.64 years High severity patients (79.33 ± 61.94 months); *N* = 6 Age: 25.5 ± 5.99 years			
	**Control group**			
	*N* = 20 (14 F/6 M) Age: 21.7 ± 3.64 years			
Pallegama et al., 2004 Cross-sectional	**Experimental group**	Masticatory muscle pain with/without DID, tenderness over the masseter and/or temporalis muscles on either side	**Sensory-motor impairments** Resting EMG activity	**Sensory-motor impairments** All TMD groups had a significantly higher resting EMG activity compared to the control group. Myogenous TMD patients with painful muscles had higher resting EMG activity in comparison with myogenous TMD patients without pain
	*N* = 38 (22 F/16 M) Age: 29 ± 10.3 years Myogenous TMD patients; *N* = 8 Myogenous and DID TMD patients; *N* = 30			
	**Control group**			
	*N* = 41 (27 F/14 M) Age: 27.3 ± 8.2 years			
Braun, 1991 Cross-sectional	**Experimental group**	A primary complaint of jaw pain and/or jaw dysfunction, at least 6 months of daily headaches and neck pain	**Cervical spine alignment** Head posture (tragus-C7-horizontal plane) using photographs	**Cervical spine alignment** Forward head position and decreased flexibility in head retraction were more frequent in symptomatic women compared to asymptomatic women
	TMD with concurrent headache and neck pain patients; *N* = 9 (F) Age: 38.11 ± 6.95 years			
	**Control group**			
	*N* = 40 (20 F/20 M) Age: F: 28.40 ± 9.29 years M: 29.00 ± 4.39 years			
Lee et al., 1995 Cross-sectional	**Experimental group**	The chief complaint related to pain in the masticatory muscles, the pain increased with jaw movement and function, tenderness caused by digital palpation in the masseter and temporalis muscles, and a maximum comfortable interincisal opening of <40 mm	**Cervical spine alignment** Head posture (tragus-C7-horizontal plane, eye-tragus-C7 angle, and ear-vertical plumb line) using photographs, camera, and ruler	**Cervical spine alignment** The angle tragus-C7-horizontal plane was smaller in the TMD group compared to the control group. The ear-vertical plumb line and eye-tragus-C7 angle showed no significant differences between groups
	Myogenous or mixed TMD patients; *N* = 33 (30 F/3 M) Age: 31.4 ± 10.1 years			
	**Control group**			
	*N* = 33 (30 F/3 M) Age: 31.4 ± 10.1 years			

AAOP: American Academy of Orofacial Pain; AROM: active range of motion; CCFT: craniocervical flexor test; CF-PDI: craniofacial pain and disability inventory; CMD: craniomandibular disorders; CSD: cervical spine disorders; DID: disc interference disorders; EMG: electromyography; F: females; FRT: flexion-rotation test; HCA: high cervical angle; ICM: index of cervical mobility; JDI: jaw disability index; JFS: jaw function scale; LDF-TMDQ: limitations of daily functions in TMD questionnaire; LDF-TMDQ/JFS: limitations of daily functions in TMD questionnaire/jaw function scale; M: males; MFIQ: mandibular functional impairment questionnaire; NDI: neck disability index; NEMET: neck extensor muscle endurance test; PDQ: pain disability questionnaire; PPT: pressure pain threshold; PROM: passive range of motion; RDC/TMD: Research Diagnostic Criteria for Temporomandibular Disorders; ROM: range of motion; TMD: temporomandibular disorders; TMJ: temporomandibular joint; TMJ ID: internal derangement temporomandibular joint; VAS: visual analog scale.

**Table 2:** Quality appraisal case-control studies.

Case-Control Studies	S1: Adequate Case Definition	S2: Representativeness of Cases	S3: Selection of Controls	S4: Definition of Controls	Ca: Controlled for Age	Cb: Controlled for Additional Factor	E1: Ascertainment of Exposure	E2: Same Method for Cases and Controls	E3: Non-Response Rate	Total	%
Da Costa etal., 2015 [47]	★			★	★		★	★		5/9	56
Raya et al., 2017 [61]	★				★	★				3/9	33
Armijo-Olivo et al., 2011 (a) [40]			★							1/9	11

S = selection; C = comparability; E = exposure.

**Table 3:** Quality appraisal cross-sectional studies.

Cross-Sectional Studies	S1: Representativeness of Exposed Cohort *	S2: Selection of Non-Exposed Cohort	S3: Ascertainment of Exposure*	S4: Outcome of Interest not Present at Start	Ca: Study Controls for Age/Gender	Cb: Study Controls for Additional Factor	O1: Ax of Outcome*	O2: Long Enough Follow-up	O3: Adequate Follow-up	Total	%
Gil Martínez et al., 2017 [52]			★				★			2/3	67
Thorp et al., 2019 [64]			★							1/3	33
Gil-Martínez et al., 2016 [51]			★				★			2/3	67
Silveira et al., 2015 [63]			★				★			2/3	67
Bragatto et al., 2016 [43]			★				★			2/3	67
Coskun et al., 2018 [46]			★							1/3	33
Greghi et al., 2018 [53]			★				★			2/3	67
Monticone et al., 2019 [58]										0/3	0
López de Uralde-Villanueva et al., 2015 [57]			★				★			2/3	67
Armijo-Olivo et al., 2010 (a) [36]			★							1/3	33
Silveira et al., 2014 [63]			★				★			2/3	67
Visscher et al., 2002 [66]			★				★			2/3	67
Armijo-Olivo et al. 2010 (b) [37]			★				★			2/3	67
Armijo-Olivo et al., 2012 [39]			★				★			2/3	67
De Laat et al., 1998 [49]							★			1/3	33
Ferreira et al., 2019 [50]			★				★			2/3	67
Grondin et al., 2015 [54]			★				★			2/3	67
von Piekartz et al., 2016 [67]			★				★			2/3	67
Iunes et al., 2009 [55]			★				★			2/3	67
Armijo-Olivo et al., 2010 (c) [38]			★				★			2/3	67
Armijo-Olivo et al., 2011 (b) [41]			★				★			2/3	67
Bevilaqua-Grossi et al., 2007 [42]			★							1/3	33
Clark et al., 1987 [45]			★							1/3	33
De Farias et al., 2010 [48]			★				★			2/3	67
Uritani et al., 2014 [65]			★							1/3	33
Munhoz et al., 2004 [59]			★							1/3	33
Pallegama et al., 2004 [60]			★							1/3	33
Braun 1991 [44]										0/3	0
Lee et al., 1995 [56]										0/3	0

S = selection; C = comparability; O = outcome.
